# Development of High-Resolution Melting Curve Analysis for rapid detection of *SEC23B* gene mutation causing Congenital Dyserythropoietic Anemia type II in Indian population

**DOI:** 10.1186/s13052-023-01493-w

**Published:** 2023-07-16

**Authors:** Arati Nandan Saptarshi, Rashmi K. Dongerdiye, Tejashree Anil More, Prabhakar S. Kedar

**Affiliations:** Department of Haematogenetics, ICMR- National Institute of Immunohaematology, 13th Floor, New Multi Storeyed Building, KEM Hospital Campus, Parel, Mumbai, 400012 India

**Keywords:** Congenital dyserythropoietic anemia, High-resolution melting curve, Sanger sequencing

## Abstract

**Background:**

Congenital dyserythropoietic anemias (CDAs) are a very rare and heterogeneous group of disorders characterized by ineffective erythropoiesis. CDA II is caused by mutations in the *SEC23B* gene. The most common mutation reported in India is c.1385 A > G, p.Y462C. There is no simple and cost-effective confirmatory diagnostic test available for CDA, and therefore, many patients remain undiagnosed. High-resolution melting curve (HRM) analysis is a polymerase chain reaction (PCR) based technique applied to identify genetic differences and scan nucleic acid sequences. HRM can be used to rapidly screen the common mutation causing CDA II in the Indian population. Thus, we studied the use of High-Resolution Melting Curve Analysis to detect common mutation causing CDA II in the Indian population.

**Method:**

11 patients having *SEC23B* (Y462C) mutation causing CDA II are considered for this study. HRM was used to check the presence of Y462C mutation. To verify the accuracy of the HRM analysis, we compared HRM results with the results of Sanger sequencing. This helped us to confirm the diagnosis.

**Results:**

We have described the clinical, hematological, and genetic data of eleven patients suffering from CDAII. According to HRM and Sanger sequencing, a homozygous *SEC23B* (Y462C) mutation was present in all patients, whereas a heterozygous Y462C mutation was present in their parents.

**Conclusion:**

Our data showed that High-Resolution Melting (HRM) analysis could be used to rapidly screen common *SEC23B* mutation that causes CDA II in the Indian population.

## Background

Congenital dyserythropoietic anemias (CDAs) are a rare and heterogeneous group of disorders. CDAs are characterized by ineffective erythropoiesis, morphological abnormalities of erythroblasts (bi/multinucleated erythroblast, internuclear bridges, erythroid hyperplasia), haemolysis. Depending on erythroid maturation and precursors involved, three major types of CDA (I, II, III) are identified. Other variants of CDA like transcription factor-related CDA include CDA type IV and X-linked thrombocytopenia with or without dyserythropoietic anemia (XLTDA). The CDA associated with Majeed syndrome is also known. The causative genes are *CDAN1*, *C15ORF41*, *SEC23B*, *KIF23*, *KLF1*, *GATA1*, and *LPIN2* [1]. Patients suffering from CDA are characterized by anemia of variable degree, pallor, recurrent jaundice, hepatomegaly, splenomegaly, and gall stones. In some cases, patients are transfusion-dependent, while in some cases, transfusion is less frequently required, and patients are asymptomatic [2].

Congenital Dyserythropoietic Anemia type II (OMIM: 224100) is the most common type of CDA. The genetic defect of CDAII is due to mutations in the *SEC23B* gene (encoding COPII) [3]. *SEC23B* gene (OMIM: 610512) is located on chromosome 20p11.23. It is involved in providing instructions for the making of one component of coat protein complex II (COPII). COPII is a large group of interacting proteins that function in the formation of vesicles. Vesicles are small sac-like structures involved in the transportation of proteins and other materials in cells. COPII triggers the formation of vesicles in the endoplasmic reticulum (ER). It plays a vital role in protein processing and transportation. COPII vesicles carry proteins that are destined to be secreted. Endoplasmic reticulum (ER)-to-Golgi trafficking is disturbed due to abnormalities in the *SEC23B* gene. This affects different glycosylation pathways and ultimately accounts for the cellular phenotype observed in CDAII [4].

*SEC23B* mutations are inherited in an autosomal recessive pattern. So far, only 16 patients of congenital dyserythropoietic anemia type II from India have been described in the literature: 6 of the patients were diagnosed based on bone marrow light microscopy [5], and 10 North Indian patients suspected of CDAII based on the clinical history and bone marrow light microscopy were confirmed to have p.Y462C mutation in exon 12 of *SEC23B* gene by molecular characterization using Sanger sequencing [6, 7]. There is a possibility of more patients suffering from CDA in the Indian population. But the data is not available because of difficulties in diagnosis. The current methods used for diagnosis include light and/or electron microscopic analysis of bone marrow aspirate[8], SDS-PAGE and molecular confirmation is carried out using next-generation sequencing (NGS) and Sanger sequencing. Next-generation sequencing and Sanger sequencing are time-consuming, laborious and comparatively costly methods. For the identification of common mutations other techniques like restriction fragment length polymorphism, high-resolution melting curve analysis can be used. More than 141 mutations have been identified in the *SEC23B* gene that cause CDA II. The most common mutation in the Indian population is c.1385 A > G, p.Y462C [9]. Many patients with CDA remain undiagnosed because of a lack of simple, confirmatory diagnostic methods.

Biochemical methods such as Eosin-5’-Maleimide assay (EMA) followed by an anti-CD44 antibody binding test, along with bone marrow microscopy and molecular tests, are found to be helpful for the diagnosis [10]. The Eosin-5’-Maleimide assay (EMA) is used for the diagnosis of red cell membrane protein defects. Patients suffering from CDA II have hypo-glycosylation of band 3 of the red cell membrane. Therefore, they show decreased mean channel fluorescence (MCF) in the EMA test. Thus, many cases of CDA II are misdiagnosed as cases of membrane protein defects like hereditary spherocytosis (HS). In CDA II patients, anti-CD44 antibody binding to red blood cells is raised compared to normal healthy individuals and HS patients [10, 11]. We used the Eosin-5’-Maleimide assay (EMA) by flow cytometry to diagnose hereditary spherocytosis and CDAII. We have further used an anti-CD44 antibody binding assay to suspect the diagnosis of CDA II in patients who have shown low mean channel fluorescence in the EMA test. Sanger sequencing was used for the confirmation of diagnosis and the utility of High-Resolution Melting (HRM) curve analysis was checked to identify the common mutation.

High-Resolution Melting (HRM) curve analysis is a polymerase chain reaction (PCR) based technique applied to identify genetic differences and scan nucleic acid sequences. The properties of DNA such as length, sequence, composition, GC content, and heterozygosity are responsible for the melting curve. Significant information about the genotype can be obtained by these melting curve profiles, which allow analysis of mutations and polymorphisms. The precision and accuracy of the HRM method are dependent on the fluorescent dye used along with the instrument and software in the analysis [12]. HRM analysis gives reproducible and rapid results though not used in routine clinical practice [13]. In this study, we have developed the HRM method to detect common *SEC23B* gene mutation causing CDA II in the Indian population.

## Methodology

### Blood collection, measurement of hematological parameters, and case history

A total of 11 suspected patients of CDA were referred to our haematogenetics laboratory of the National Institute of Immunohematology (NIIH), KEM Hospital, Mumbai, India. All patients were of Indian origin and were from genetically unrelated families. The available family members of 11 patients were included in this study. With the approval of ICMR- National Institute of Immunohematology- Ethics Committee guidelines, we took written informed consent from all the patients and their family members. We reviewed the patients’ family history and clinical, morphological, and laboratory data. The CDA was suspected based on clinical findings and in some patients based on bone marrow analysis. The complete blood count (CBC) using the automated haematology analyzer (Sysmex XN, Sysmex corporation) and morphological examination of red blood cells was performed. The confirmation of the diagnosis of CDA was done by molecular analysis using Sanger sequencing and newly developed High-Resolution Melting (HRM) curve analysis.

### Eosin 5’ maleimide binding assay (EMA)

5µL washed packed red blood cells from all patients and healthy controls were mixed with 25µL of EMA dye (0.5 mg/mL in PBS) and incubated for an hour in the dark at room temperature. Phosphate Buffer Saline–bovine serum albumin (PBS-BSA) solution was used to wash EMA dye-labeled RBCs. Washed RBCs were then resuspended in 500 µL of PBS-BSA solution. 50µL of RBCs labelled with EMA were resuspended in 700 µL of 0.5% PBS-BSA solution for analysis on BD FACSAria™ Fusion Flow Cytometer. Results were expressed in Mean Channel Fluorescence (MCF) as described earlier for the diagnosis of red cell membrane protein defect [14]. As described in the literature, in this study also low or borderline MCF was observed in CDA II patients [10, 11].

### CD44 antibody binding assay

10µL of whole blood was diluted with PBS (1:150 dilution). 30µL of this diluted sample was incubated with the titrated volume of anti-CD44 antibody (BD Biosciences) in the dark at room temperature for 20 min. CD44 labelled RBCs were then washed and resuspended in 300 µL of PBS and used for analysis on BD FACSAria™ Fusion Flow Cytometer, and results were expressed in MCF as described earlier [10].

### Isolation of DNA and mutational analysis of *SEC23B* gene using sanger sequencing

FlexiGene kit was used for the isolation of genomic DNA from peripheral blood. Amplification of DNA for exon 12 of *SEC23B* gene was carried out using oligonucleotides primers [Exon-12F:5’TAGCCTGCCCTACTCAGTCA 3’ and Exon-12R: 5’ TGACTCCGACTAAACCCTTG 3’]. The primers are made using NCBI-Primer BLAST software (https://www.ncbi.nlm.nih.gov/tools/primer-blast/). The PCR conditions were: 95^0^ C for 5 min (94^0^ C for 20 sec, 58^0^ C for 20 sec, 72^0^ C for 20 sec) for 35 cycles and a final extension at 72^0^ C for 5 min. QIAquick Gel Extraction Kit was used to purify the amplicons further, and purified products were used for mutation analysis. ABI PRISM BigDye Terminator Cycle Sequencing Ready Kit was used for sequencing purified PCR products, and they were run on an ABI Prism 3730xl Genetic Analyzer (Applied Biosystems, USA). CHROMAS software v2.6.6 was used to analyze the obtained chromatograms.

### High-resolution melting (HRM) analysis for screening of *SEC23B*-Y462C mutation

Real-time PCR (Applied Biosystems StepOne™ Real-Time PCR) was used to perform High-Resolution Melting (HRM) assay for the screening of *SEC23B*-Y462C mutation. 10.0 µL of HRM Master Mix manufactured byMeltDoctor™, 0.4µL of forward primer (10 pmoles) [Exon-12F:5’TAGCCTGCCCTACTCAGTCA 3’] and 0.4µL of reverse primer (10 pmoles)[Exon-12R: 5’TGACTCCGACTAAACCCTTG 3’], 2.0 µL of DNA having concentration 50–100 ng,7.2 µL of HPLC water making a reaction mixture of 20.0 µLwas used for the assay. The cycling conditions were as follows: denaturation at 95^0^ C for 10 min (95^0^ C for 15 sec, 60^0^ C for 1 min) for 40 cycles. The temperature was raised to 97^0^ C following continuous fluorescence detection of 25 acquisitions for the development of melting curves [12]. StepOne Software v2.3 was used for the completion of all steps by using HRM Software v3.0.1 to carry out an analysis.

## Results

### Clinical data

**Fig. 1:**
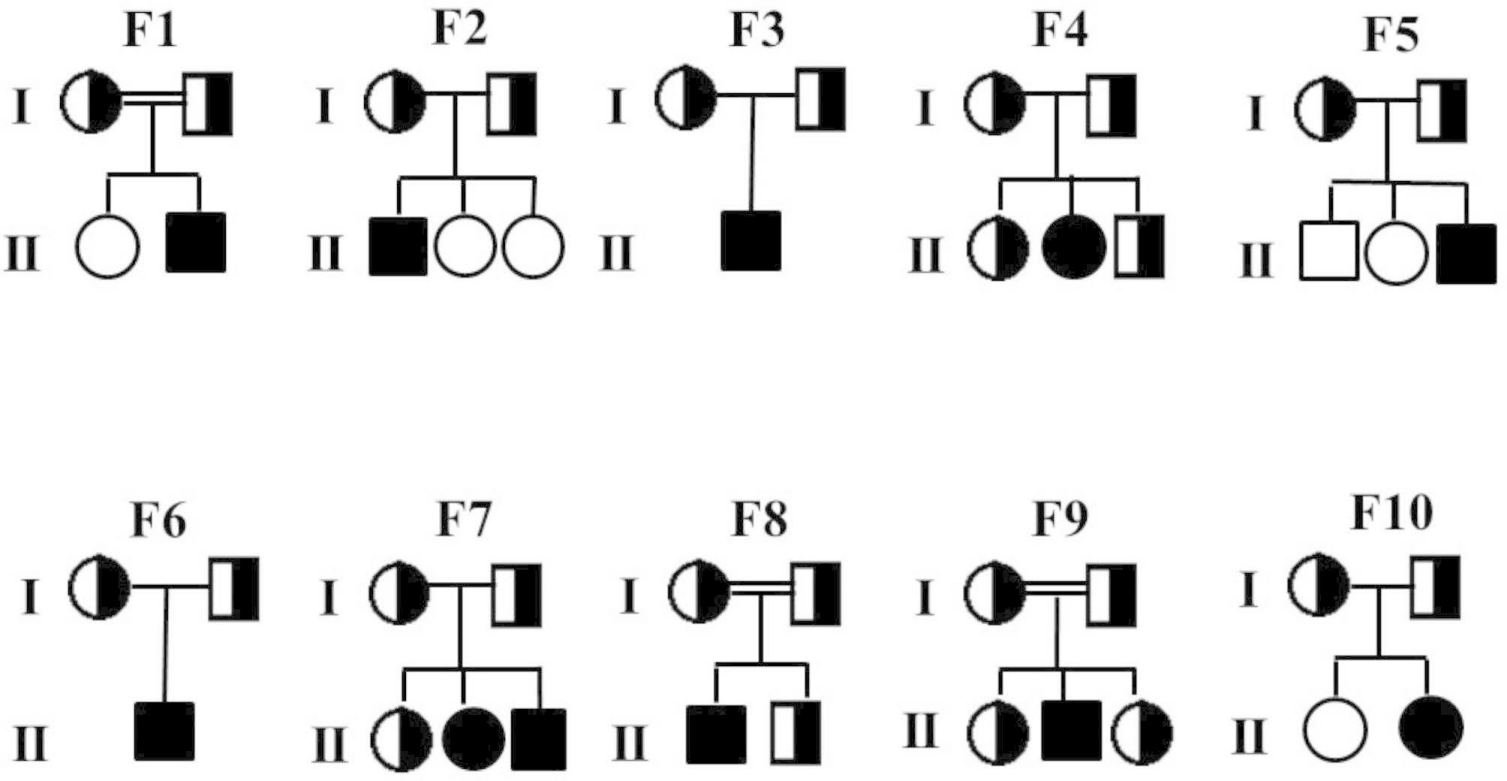
Pedigree structure and segregation analysis of families affected by congenital dyserythropoietic anemia (CDA). Individuals affected with CDAII p.Y462C homozygous mutation are indicated by a black-filled circle (females) or square (males). Heterozygous individuals are indicated by half-filled circles (females) and half-filled squares (males)

11 CDA patients (8 males and 3 females) were included in our study. Their ages ranged from 3 months to 33 years. The median age was 12 years. Pedigree structure and segregation analysis of families affected by congenital dyserythropoietic anemia (CDA) are shown in (Fig. 1**)**. The hematological, biochemical, and clinical manifestations in these patients are summarized in (Table 1**)**. The clinical picture of all 11 patients showed recurrent jaundice, severe anemia, and hepatosplenomegaly of varying degrees, and most of the patients presented with weakness, fatigue, and recurrent stomach aches. Serum ferritin and lactate dehydrogenase (LDH) was increased in all the patients. Patients’ peripheral blood smear showed hypochromic cells, teardrop cells, and a few spherocytes, and the bone marrow smear showed erythroid hyperplasia, few bi &multinucleated erythroblasts, and mild dyserythropoiesis. Index case from family 1 had short stature and cushingoid features. The index case from family 2 had antinuclear antibody (ANA) positive, and the index case of family 8 had a congenital disorder of glycosylation along with congenital dyserythropoietic anemia.

**Table 1:** Haematological, Biochemical and Clinical Profile of 11 Patients with CDA type II

Family	Proband	Age at diagnosis/ Sex	RBC (x10^6^/ul)	Hb (g/dL)	HCT (%)	MCV (fl.)	MCHC (g/dL)	RDW (%)	Retic (%)	BM-LM	Presenting Symptoms	EMA (MCF)
F1	II – 2	5Y/M	1.89	4.3	13.8	73	31.2	29.7	3	Erythroid hyperplasia, few bi & multinucleated erythroblasts, occasional karyorrhectic forms, mild dyserythropoiesis	Chronic anemia, Hepato-Splenomegaly	849
F2	II – 1	33Y/M	2.16	5.8	17.3	80.1	33.5	23	0.9	Erythroid hyperplasia, binucleated erythroblasts, dyserythropoiesis	Pallor, Icterus, anemia Cholelithiasis, haemolytic facies	593
F3	II – 1	3 m/M	2.7	7.5	20.7	76.7	36.2	15.2	9	Not Done	Severe anemia, hepato-splenomegaly, neonatal jaundice	886
F4	II – 2	9 Y/ F	2.64	6.9	19.1	72.3	36.1	27.3	8	Erythroid Hyperplasia, binucleated erythroblasts	Chronic anemia, Hyperbilirubinemia	630
F5	II – 3	12Y/M	2.66	7	21.5	80.8	32.6	30	4	Not Done	Severe anemia, Icterus	995
F6	II – 1	9Y/ M	1.65	6.2	18.3	110.9	33.9	20.7	2.4	Erythroid Hyperplasia, a large number of bilobed erythroid cells, some nuclear bridging	Severe anemia, Neonatal Jaundice, splenomegaly	850
F7	II – 3	13Y/M	3.33	8.3	24.4	73.3	34	24.8	1.3	Not Done	Chronic haemolytic anemia, Icterus, Pallor, hepatosplenomegaly	780
	II – 2	18Y/F	2.94	8.8	24.1	82	36.5	22.1	1.6	Erythroid hyperplasia, mild dyserythropoiesis, multinucleated erythroblasts, karyorrhexis, nuclear bridging	Severe anemia, recurrent jaundice, pallor, chipmunk facies	856
F8	II − 3	15Y/M	3.68	9.9	32	87	30.9	26	5	Not Done	Anemia, pallor, icterus, moderate splenomegaly	754
F9	II − 2	12Y/M	3.0	7.4	22.5	75	32.9	21.7	0.1	Dyserythropoietic normoblasts	Severe pallor, severe anemia, neonatal jaundice, splenomegaly	918
F10	II − 2	6 m/F	1.8	4	12.8	71.1	31.3	29.1	2.8	Not Done	Severe pallor, anemia, hemangioma	926

**Abbreviations**: BM-LM: Bone Marrow analysis using Light Microscopy, EMA: Eosin-5’-Maleimide, MCF: Mean Channel Fluorescence

### Flow cytometric analysis of EMA binding assay and anti-CD44 antibody binding assay

**Fig. 2:**
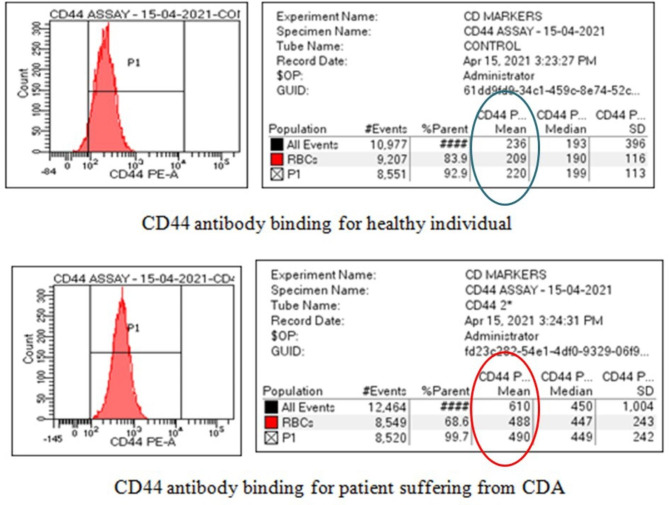
The RBCs of CDA II patients show an increased level of CD44 (MCF: 490) as compared to the RBCs of a normal healthy individual (MCF: 220)

A significant decrease in MCF using EMA binding assay was observed in 11 CDAII patients as shown in (Table 1**)**. (Normal range: 900–1300 MCF units). The anti-CD44 antibody binding test using flow cytometric analysis was performed; positive results were obtained based on elevated mean channel fluorescence values compared to normal healthy individuals. All patients with CDAII showed almost twice MCF in the anti-CD44 antibody binding test than normal healthy individuals (Fig. 2).

### Sanger sequencing for *SEC23B*-Y462C mutation

**Fig. 3:**
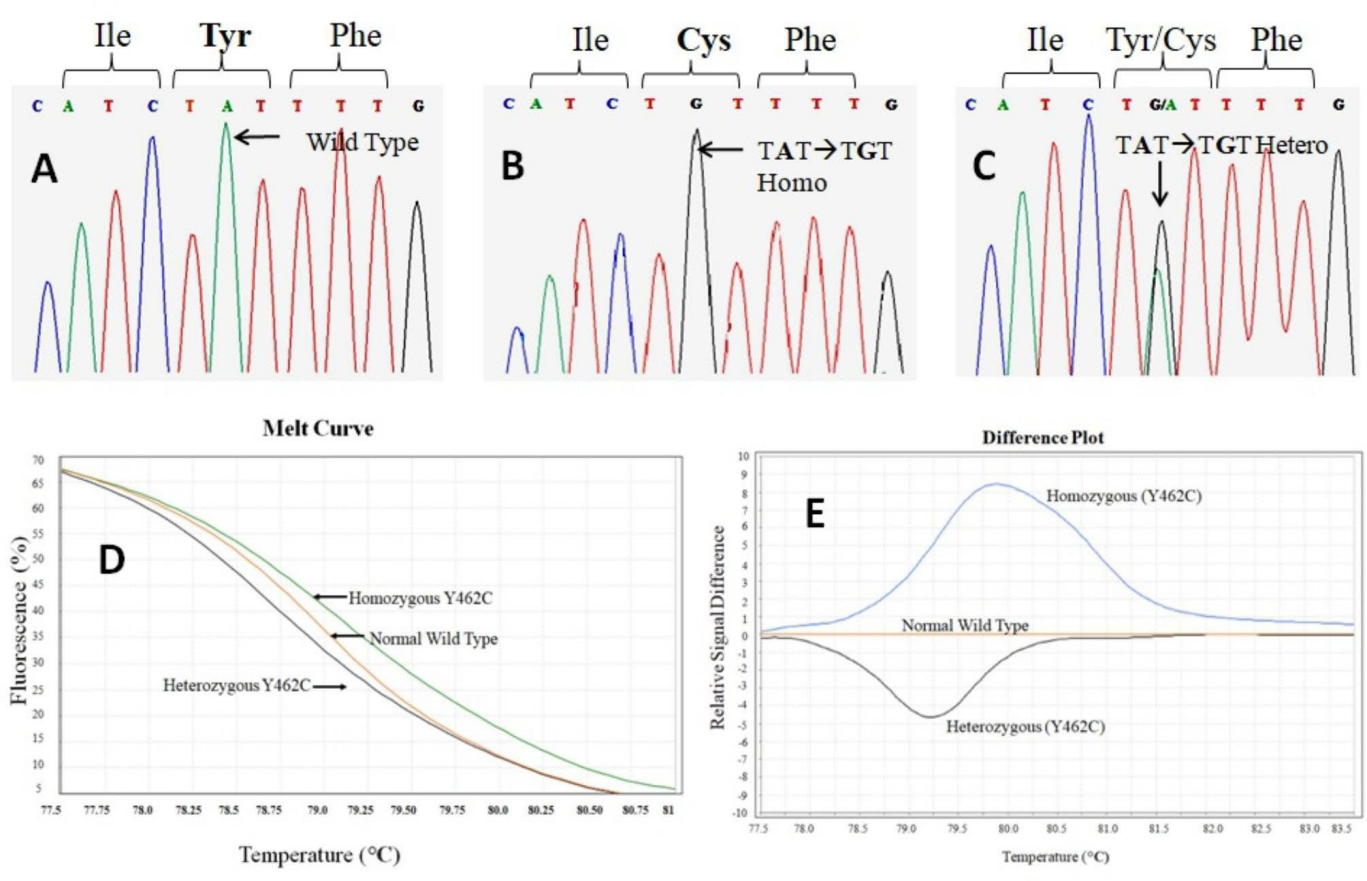
Sanger sequencing of an amplified DNA fragment of exon 12 of *SEC23B* gene (**A**) Normal (Wild Type) (**B**) TGT/TGT (Homozygous) (**C**) TAT/TGT (Heterozygous for CDA having normal phenotype) and HRM profile for genotypic differentiation. (**D**) Melt curve showing homozygous (Y462C), heterozygous, and normal wild type. (**E**) Difference plot showing homozygous (Y462C), heterozygous and normal wild type

The Sanger sequencing was performed for confirmed diagnosis ofCDAII in patients and also to verify the results obtained by HRM analysis. Sanger sequencing revealed substitution of nucleotide in exon 12 in *SEC23B* gene at codon 462 (T**A**T →T**G**T), which corresponds to homozygous Tyr462Cys (Y462C) mutation in all patients (Fig. 3B), and their parents and some of the family members showed heterozygous p.Y462C genotype for CDAII having normal phenotype (Fig. 3C) (Table 2**)**. There was no other mutation present in the *SEC23B* gene in any of the patients.

**Table 2:** Genotyping of *SEC23B –* Y462C mutation in patients with CDA and family members using HRM analysis and Sanger sequencing

Family	Number	Relationship	Clinical Diagnosis	Nucleotide change		Amino acid change
				Sanger Sequencing	HRM analysis	
F1	I – 1	Mother	Normal	TAT/TGT	TAT/TGT	N/Y462C
	I – 2	Father	Normal	TAT/TGT	TAT/TGT	N/Y462C
	II – 1	Daughter	Normal	TAT/TAT	TAT/TAT	N/N
	II − 2	Son	**CDA**	TGT/TGT	TGT/TGT	Y462C/Y462C
F2	I – 1	Mother	Normal	TAT/TGT	TAT/TGT	N/Y462C
	I – 2	Father	Normal	TAT/TGT	TAT/TGT	N/Y462C
	II – 1	Son	**CDA**	TGT/TGT	TGT/TGT	Y462C/Y462C
	II – 2	Daughter	Normal	Not Done	Not Done	Not Done
	II − 3	Daughter	Normal	Not Done	Not Done	Not Done
F3	I – 1	Mother	Normal	TAT/TGT	TAT/TGT	N/Y462C
	I – 2	Father	Normal	TAT/TGT	TAT/TGT	N/Y462C
	II − 1	Son	**CDA**	TGT/TGT	TGT/TGT	Y462C/Y462C
F4	I – 1	Mother	Normal	TAT/TGT	TAT/TGT	N/Y462C
	I – 2	Father	Normal	TAT/TGT	TAT/TGT	N/Y462C
	II- 1	Daughter	Normal	TAT/TGT	TAT/TGT	N/Y462C
	II – 2	Daughter	**CDA**	TGT/TGT	TGT/TGT	Y462C/Y462C
	II − 3	Son	Normal	TAT/TGT	TAT/TGT	N/Y462C
F5	I – 1	Mother	Normal	TAT/TGT	TAT/TGT	N/Y462C
	I – 2	Father	Normal	TAT/TGT	TAT/TGT	N/Y462C
	II – 1	Son	Normal	TAT/TAT	TAT/TAT	N/N
	II – 2	Daughter	Normal	Not Done	Not Done	Not Done
	II − 3	Son	**CDA**	TGT/TGT	TGT/TGT	Y462C/Y462C
F6	I – 1	Mother	Normal	TAT/ TGT	TAT/ TGT	N/Y462C
	I – 2	Father	Normal	TAT/ TGT	TAT/ TGT	N/Y462C
	II − 1	Son	**CDA**	TGT/ TGT	TGT/ TGT	Y462C/Y462C
F7	I – 1	Mother	Normal	TAT/ TGT	TAT/ TGT	N/Y462C
	I – 2	Father	Normal	TAT/ TGT	TAT/ TGT	N/Y462C
	II – 1	Daughter	Normal	TAT/ TGT	TAT/ TGT	N/Y462C
	II – 2	Daughter	**CDA**	TGT/ TGT	TGT/ TGT	Y462C/Y462C
	II − 3	Son	**CDA**	TGT/ TGT	TGT/ TGT	Y462C/Y462C
F8	I – 1	Mother	Normal	TAT/ TGT	TAT/ TGT	N/Y462C
	I – 2	Father	Normal	TAT/ TGT	TAT/ TGT	N/Y462C
	II – 1	Son	**CDA**	TGT/ TGT	TGT/ TGT	Y462C/Y462C
	II − 2	Son	Normal	TAT/ TGT	TAT/ TGT	N/Y462C
F9	I – 1	Mother	Normal	TAT/ TGT	TAT/ TGT	N/Y462C
	I – 2	Father	Normal	TAT/ TGT	TAT/ TGT	N/Y462C
	II – 1	Daughter	Normal	TAT/ TGT	TAT/ TGT	N/Y462C
	II – 2	Son	**CDA**	TGT/ TGT	TGT/ TGT	Y462C/Y462C
	II − 3	Daughter	Normal	TAT/ TGT	TAT/ TGT	N/Y462C
F10	I – 1	Mother	Normal	TAT/ TGT	TAT/ TGT	N/Y462C
	I – 2	Father	Normal	TAT/ TGT	TAT/ TGT	N/Y462C
	II – 1	Daughter	Not Done	Not Done	Not Done	Not Done
	II − 2	Daughter	**CDA**	TGT/ TGT	TGT/ TGT	Y462C/Y462C

#### HRM screening of *SEC23B*-Y462C mutation

Results of HRM analysis showed the presence of a prominent peak of homozygous c.1385 A > G, (p.Y462C) mutation in all index cases. Analysis was performed by HRM analysis software v3.0.1. HRM profile for genotypic differentiation in the melting curve showing homozygous (Y462C), heterozygous, and wild type is shown in (Fig. 3D), and a difference plot showing homozygous (Y462C), heterozygous and wild type is shown in (Fig. 3E). The high-resolution melting curves and difference plots of patients showed the homozygous mutation, and their parents showed the heterozygous mutation. On the RT-PCR screen of HRM analysis, all patients showed a prominent peak of homozygous Y462C mutations, and their parents showed the heterozygous peak of Y462Cmutation.

## Discussion

Congenital Dyserythropoietic Anemia (CDA) is a very rare disease. The diagnosis of CDA is difficult because of the lack of detailed diagnostic procedures. It is mainly diagnosed when other causes of hemolytic anemia are ruled out and this often delays the diagnosis [2]. Thus, many patients suffering from CDA have no precise diagnosis, because of which effective patient management and genetic counseling are often delayed or missed as it happens in most of the rare disorders [15]. Currently, morphological analysis of bone marrow aspirate, molecular characterization using next-generation sequencing, and Sanger sequencing are considered to be confirmatory diagnostic tests. These tests are costly and time-consuming. In the Indian population, many patients suffering from CDAII show c.1385 A > G, p.Tyr462Cys mutation in the *SEC23B* gene. Thus, in this study, we have developed an HRM analysis method to detect c.1385 A > G, p.Y462C mutation in the *SEC23B* gene causing CDA II. High-resolution melting (HRM) curve analysis is a polymerase chain reaction-based technique that identifies genetic differences and scans nucleic acid sequences. The *SEC23B* variations are currently detected using conventional methods like PCR-SSCP, allele-specific amplification, Sanger sequencing and next-generation sequencing [8]. All of these methods are lengthy and costly. They involve open tube assays, which need processing after PCR. The post-PCR processing includes electrophoresis and/or sequencing. This method makes it very laborious. Further, PCR products need to be transferred for further analysis. In this, there is a high risk of contamination. For Sanger sequencing and NGS, specific instruments are required which are not available in all the laboratories. Wittwer and his colleagues developed a high-resolution melting curve analysis of amplified products using dsDNA dye in the late 1990s [16, 17]. The advantage of the HRM method is that the same reaction tube is used for amplification and detection steps. This eliminates possible post-PCR contaminations that can occur in above mentioned conventional methods. For laboratories with a large number of samples for analysis or laboratories with less funding, it is challenging to do DNA Sanger sequencing even though now the cost for the same is relatively low. After isolating and extracting genomic DNA, the HRM analysis takes only a few hours to completion, and visually comparing the melting curves on the RT-PCR screen is sufficient for diagnosis. This makes the process fast and has high throughput. Analysis using a High-Resolution Melting curve is economically beneficial as the technique has minimal requirements[18]. The cost of saturating DNA dye is less than PCR reagents, although the thermal cycler capable of measuring real-time fluorescence is required. Using the HRM method, identifying single-base changes that cause heterozygosity is possible [19]. In this study, HRM analysis showed homozygous Y462C mutation in all CDA II patients, and their parents and a few family members showed heterozygous Y462C mutation. We used Sanger sequencing for confirmation of the diagnosis of CDA II and verification of the results obtained by HRM analysis. This confirmed the efficiency of HRM analysis in screening common *SEC23B* mutation in CDA II patients in the Indian population. In all the cases, diagnosis and confirmation were carried out using biochemical tests – low MCF in EMA and raised CD44 antibody binding and molecular tests – HRM and Sanger sequencing. Thus, patients suspected to have CDA II based on clinical features like moderate to severe anemia, normal to slightly high reticulocyte count, increased ferritin and LDH levels could be diagnosed on the basis of EMA followed by CD44 antibody binding and screening for common mutation in the Indian population using HRM analysis. All these tests are cost-effective and require less time compared to currently used other methods like next-generation sequencing and Sanger sequencing.

## Conclusion

Considering these advantages of HRM, we have developed a protocol for studying the common mutation (*SEC23B*-Y462C) causing congenital dyserythropoietic anemia type II in the Indian population. Our data showed that High-Resolution Melting (HRM) analysis could be used for rapid screening of common *SEC23B* mutation that causes CDAII in the Indian population. HRM technique leads to an accurate diagnosis in CDAII patients and does not need further diagnostic workup. Earlier incorporation of HRM technique for diagnosing CDAII in Indian patients will improve patient care and enable genetic counselling and prenatal diagnosis.

## Data Availability

Not Applicable, all data included in the manuscript.

## Abbreviations

CDA: Congenital dyserythropoietic anemia
HRM: High-Resolution Melting
PCR: Polymerase Chain Reaction
SDS-PAGE: Sodium Dodecyl Sulfate–Polyacrylamide Gel Electrophoresis
OMIM: Online Mendelian Inheritance in Man
COPII: Coat Protein complex II
ER: Endoplasmic Reticulum
CD44: Cluster of Differentiation 44
EMA: Eosin-5’-Maleimide Assay
PBS: Phosphate Buffer Saline
BSA: Bovine Serum Albumin
MCF: Mean Channel Fluorescence
SSCP: Single Strand Conformation Polymorphism
CDAN1: Codanin 1
C15ORF41: Chromosome 15 Open Reading Frame 41
SEC23B: SEC23 Homolog B, COPII Coat Complex Component
KIF23: Kinesin Family Member 23
KLF1: Krueppel-like factor 1
GATA1: GATA-binding factor 1- Erythroid transcription factor
LPIN2: Lipin 2
XLTDA: X-linked thrombocytopenia with or without dyserythropoietic anemia
NGS: Next Generation Sequencing
RT-PCR: Real-Time Polymerase Chain Reaction

## References

[1] Russo R, Esposito MR, Asci R, Gambale A, Perrotta S, Ramenghi U, Iolascon A (2010). Mutational spectrum in congenital dyserythropoietic anemia type II: identification of 19novel variants in SEC23B gene. Am J Hematol.

[2] Iolascon A, Andolfo I, Russo R (2020). Congenital dyserythropoietic anemias Blood.

[3] Iolascon A, Russo R, Delaunay J (2011). Congenital dyserythropoietic anemias. Curr OpinHematol.

[4] Bianchi P, Fermo E, Vercellati C, Boschetti C, Barcellini W, Iurlo A, Zanella (2009). A.Congenital dyserythropoietic anemia type II (CDAII) is caused by mutations in the SEC23Bgene. Hum Mutat.

[5] Marwaha RK, Bansal D, Trehan A, Garewal G, Marwaha N (2003). Congenital dyserythropoietic anemia: clinical and hematological profile. Indian Pediatr.

[6] Sharma P, Das R, Bansal D, Trehan A (2015). Congenital dyserythropoietic anemia type IIwithSEC23B exon 12 c.1385A→G mutation and pseudo-gaucher cells in two siblings. Hematology.

[7] Das R, Jamwal M, Aggarwal A, Sharma P, Sachdeva MUS, Bansal D, Maitra. A. Spectrum of Genetic Defects and Phenotype-Genotype Correlation in Dyserythropoietic Anemias: Bench to Bedside Approach in the Indian Scenario. Blood. 2019. 10.1182/blood-2019-126453.

[8] Heimpel H, Kellermann K, Neuschwander N, Högel J, Schwarz K (2010). The morphological diagnosis of congenital dyserythropoietic anemia: results of a quantitative analysis of peripheral blood and bone marrow cells. Haematologica.

[9] Singleton B, Bansal D, Varma N, Das R, Naseem S, Saikia UN, Ahmed (2015). M.Homozygosity mapping reveals founder SEC23B-Y462C mutations in indian congenital dyserythropoietic anemia type II. Clin Genet.

[10] Kedar P, Parmar V, Devendra R, Gupta V, Warang P, Madkaikar M (2017). Congenital dyserythropoietic anemia type II mimicking hereditary spherocytosis in indian patients withSEC23B-Y462C mutations. Ann Hematol.

[11] Singleton BK, Ahmed M, Green CA, Heimpel H, Wozniak MJ, Ranjha L, King MJ (2018). CD44 as a potential screening marker for preliminary differentiation between congenital dyserythropoietic anemia type II and hereditary spherocytosis. Cytometry B ClinCytom.

[12] More TA, Kedar PS (2021). Genotypic analysis of SLC4A1 A858D mutation in indian population associated with distal renal tubular acidosis (dRTA) coupled with hemolytic anemia. Gene.

[13] Erali M, Wittwer CT (2010). High-resolution melting analysis for gene scanning. Methods.

[14] Kedar PS, Colah RB, Kulkarni S, Ghosh K, Mohanty D. Experience with eosin-5’-maleimide as a diagnostic tool for red cell membrane cytoskeleton disorders. Clin Lab Haematol. 2003;25(6):373-6. doi: 10.1046/j.0141-9854.2003.00557.x. PMID: 14641141. 10.1046/j.0141-9854.2003.00557.x 14641141

[15] Paola Giordano G, Lassandro M, Valente AC, Molinari, Antonio Coppola (2014). Current management of the hemophilic child: a demanding interlocutor. Quality of life and adequate cost-efficacy analysis. Pediatr Hematol Oncol.

[16] Wittwer CT, Reed GH, Gundry CN, Vandersteen JG, Pryor RJ (2003). High resolution genotyping by amplicon melting analysis using LC green. Clin Chem.

[17] Yue L, Lin M, Chen JT, Zhan XF, Zhong DS, Monte-Nguba SM, Yang L (2014). Y.Rapid screening for sickle cell disease by polymerase chain reaction-high resolution melting analysis. Mol Med Rep.

[18] Montgomery JL, Sanford LN, Wittwer CT (2010). High-resolution DNA melting analysis in clinical research and diagnostics. Expert Rev Mol Diagn.

[19] Everman S, Wang SY (2019). Distinguishing Anuran species by high-resolution melting analysis of the COI barcode (COI-HRM). Ecol Evo.

